# Sexual dysfunction in women with type 2 diabetes mellitus: A single-centre cross-sectional study from Bangladesh

**DOI:** 10.20945/2359-3997000000635

**Published:** 2023-05-29

**Authors:** A. B. M. Kamrul-Hasan, Muhammad Shah Alam, Nusrat Zarin, Fatema Tuz Zahura Aalpona, Marufa Mustari, Farhana Akter, Nadia Jannat, Umme Azad, Palash Kumar Chanda, Abdur Rafi, Mohammad Jahid Hasan, Shahjada Selim

**Affiliations:** 1 Mymensingh Medical College Department of Endocrinology Mymensingh Bangladesh Department of Endocrinology, Mymensingh Medical College, Mymensingh, Bangladesh; 2 Army Medical College Cumilla Bangladesh Army Medical College, Cumilla, Bangladesh; 3 Bangladesh Institute of Health Sciences Department of Endocrinology Dhaka Bangladesh Department of Endocrinology, Bangladesh Institute of Health Sciences, Dhaka, Bangladesh; 4 Mymensingh Medical College Hospital Outpatient Department (Gyne & Obs) Mymensingh Bangladesh Outpatient Department (Gyne & Obs), Mymensingh Medical College Hospital, Mymensingh, Bangladesh; 5 Bangabandhu Sheikh Mujib Medical University Department of Endocrinology Dhaka Bangladesh Department of Endocrinology, Bangabandhu Sheikh Mujib Medical University, Dhaka, Bangladesh; 6 Chittagong Medical College Department of Endocrinology Chittagong Bangladesh Department of Endocrinology, Chittagong Medical College, Chittagong, Bangladesh; 7 BRB Hospitals Limited Department of Endocrinology Dhaka Bangladesh Department of Endocrinology, BRB Hospitals Limited, Dhaka, Bangladesh; 8 Mymensingh Medical College Hospital Department of Endocrinology Mymensingh Bangladesh Department of Endocrinology, Mymensingh Medical College Hospital, Mymensingh, Bangladesh; 9 Pi Research Consultancy Center Dhaka Bangladesh Pi Research Consultancy Center, Dhaka, Bangladesh; 10 Bangabandhu Sheikh Mujib Medical University Department of Endocrinology Dhaka Bangladesh Department of Endocrinology, Bangabandhu Sheikh Mujib Medical University, Dhaka, Bangladesh

**Keywords:** Prevalence, sexual dysfunction, FSFI, women, diabetes mellitus, Bangladesh

## Abstract

**Objective:**

Sexual dysfunction among women with diabetes is a common but neglected health issue worldwide. The objective of the present study was to investigate the prevalence of sexual dysfunction and its associated factors among women with type 2 diabetes mellitus (T2DM).

**Subjects and methods:**

This cross-sectional comparative study comprises 150 women with diabetes and 100 healthy women without diabetes who visited the endocrinology outpatient department of Mymensingh Medical College Hospital (MMCH). The data were collected from July to December 2019. Sexual dysfunction was assessed by the 19-item Female Sexual Function Index (FSFI). Informed consent was obtained before participation. Collected data were analysed by SPSS 26.

**Results:**

More women with diabetes than control subjects reported sexual dysfunction (79% *vs.* 72%; p = 0.864). The global FSFI score was lower among the diabetes patients than among the healthy controls (20.8 ± 7.2 *vs.* 23.7 ± 4.8; p < 0.001). Patients with T2DM scored significantly lower in the domains of desire (p = 0.04), lubrication (p = 0.01), orgasm (p = 0.01), and satisfaction (p < 0.001), but not the domain of arousal (p = 0.09). A prolonged duration of diabetes was the primary contributor to orgasm problems (adjusted odds ratio, aOR 1.3, 95% CI 1.1-1.7) and painful intercourse (aOR 1.2, 95% CI 1.1-1.5).

**Conclusion:**

Sexual problems are frequent in women with diabetes. Inclusion of sexual health in comprehensive diabetes management is crucial to address this problem as well as to improve the quality of life of female diabetes patients.

## INTRODUCTION

Sexual dysfunction among women is a common but overlooked and stigmatized health concern worldwide. It is characterized by disturbances in sexual desire and in the psychophysiological changes associated with the sexual response cycle in women (1). The report of the International Consensus Development Conference on Female Sexual Dysfunction (FSD) classified FSD into four disorders: designated desire disorders (DD), arousal disorders (AD), orgasmic disorders (OD) and pain disorders (PD) (2). Although these disorders are highly prevalent among women, detailed data are few. The estimated prevalence ranges from 25%-63%, with wide variation between Eastern and Western countries and between reproductive and postmenopausal age groups (1,3). Nevertheless, a recent meta-analysis reported that almost two-fifths of sexually active women suffer from some sort of sexual dysfunction worldwide (4). In Bangladesh, population-based data are lacking; however, centre-based data reported that 51.8% of women had one or more sexual problems (5). The exact pathophysiology and aetiology remain less understood, but the prevalence is notably higher among women with different chronic conditions, such as diabetes mellitus (68%) (6), hypertension (14 to 90%) (7), and malignant diseases (78%) (8).

Diabetes mellitus (DM) is a chronic debilitating disease affecting multiple organs with a range of long-term micro- and macrovascular complications (9). The psychological impact of diabetes is also devastating and compromises the quality of life, including the sexual health, of patients (10,11). There is hardly any conclusive evidence on the pathophysiology of sexual dysfunctions in female patients with diabetes. Some researchers hypothesize that diabetes-related vascular and nerve dysfunctions may result in impaired arousal and orgasmic sexual responses due to decreased genital blood flow, atherosclerotic damage, and endothelial dysfunction (12,13). In addition, hyperglycaemia reduces the hydration of mucous membranes in the vagina and induces a suitable environment for infections, leading to decreased lubrication and dyspareunia (12). In addition, diabetes-related complications affect psychological wellbeing and relationship status, contributing to the detrimental sexual performance of women (13,14).

Although it is well established that women with diabetes are more prone to sexual dysfunction, the prevalence shows huge disparities among countries as well as the type of diabetes. Studies have reported a prevalence from 27% to as high as 94% among women with type 1 and type 2 diabetes, respectively (15-17). However, the disparity could be explained by the difference in the sample population and the measurement tool used to detect dysfunction (6). In Bangladesh, >8.4 million, which constitutes almost 8% of the total adult population, are affected by T2DM (18). Despite the very large burden, there is little evidence on the prevalence and associated factors of sexual dysfunction among diabetes patients irrespective of sex (19). Hence, the study aimed to investigate the prevalence of sexual dysfunction and its associated factors in women with T2DM compared to women without diabetes. Understanding the epidemiology and risk factors could guide further strategies for the prevention and treatment of these patients.

## SUBJECTS AND METHODS

### Study setting and participants

The sample of this cross-sectional comparative study consisted of women who were receiving treatment from the endocrinology outpatient department of Mymensingh Medical College Hospital (MMCH). The data were collected from July 2019 to December 2019.

The sample size required for the study was calculated from the following formula: $n=\frac{z^{2}p\left(1-p\right)}{d^{2}}$ , where p = estimated prevalence of sexual dysfunction among female patients with T2DM, and d = precision of error in the prevalence estimate. A recent meta-analysis including 3892 female patients with diabetes from 25 studies reported that the overall prevalence of sexual dysfunction was 68.6% (6). Considering this information for a 95% confidence level and 10% precision of error in the prevalence estimate, the formula provided that 176 patients would be enough for the present study. Along with these patients with diabetes, we also included 100 healthy patients without diabetes for comparison. However, information on the glycaemic control (HbA1c value) of 26 T2DM patients was not available. After excluding those patients, a total of 250 individuals (150 patients with diabetes and 100 healthy controls without diabetes) were included in the study. However, the control group was not matched with the T2DM patients in the present study.

Convenience sampling according to the inclusion and exclusion criteria was used for patient recruitment in the present study. To be considered eligible for participation, subjects had to fulfil the following criteria: women aged 18-45 years who had been diagnosed with T2DM for at least six months, defined as HbA1c of greater than or equal to 6.5% or fasting blood glucose of greater than or equal to 126 mg/dL and/or two-hour blood glucose of greater than or equal to 200 mg/dL in OGTT, and evidenced by receipt of at least one anti-diabetes medication or prescription from a registered physician or possession of a reliable lab report supporting the diagnosis. Healthy women who were never diagnosed with T2DM and with fasting plasma glucose < 100 mg/dL during hospital visits who were willing to participate in the study were eligible for inclusion in the comparative group. Exclusion criteria were as follows: a diagnosis of type 1 diabetes (T1DM); current pregnancy or lactation; acute illness; any psychiatric disorder; dementia; use of antipsychotics, antidepressants or any psychotropic medications; use of medications that may impair memory or cognition; recent (within six months) severe complications of diabetes, such as vascular events; dialysis treatment or having a chronic debilitating illness (such as malignancy or autoimmune diseases); and sexually inactivity in the preceding six months.

### Data collection procedure

A total of 150 women agreed to take part in the study. All women were interviewed by two trained physicians. A structured pretested questionnaire was used to collect detailed information on the participants. The questionnaire was reviewed by two consultant endocrinologists and pretested among 20 patients with diabetes for further linguistic clarification; these patients were excluded from this study. The questionnaire had two parts: (i) sociodemographic and diabetes-related information and (ii) assessment of sexual dysfunction by the Female Sexual Function Index (FSFI).

**Figure 1:**
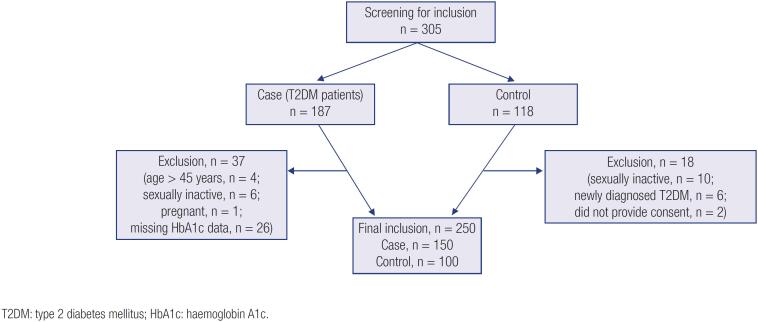
Flowchart of inclusion of patients and controls.

### Measures

#### Sociodemographic and diabetes-related information

The sociodemographic variables included patients’ age, residence, education, type of family, occupation, duration of marriage, husbands’ age, number of children, age of last child, and anthropometric measurements. Clinical history included menstrual history and history of comorbid hypertension. Standing height was measured to within 1 mm without shoes using a wall-mounted stadiometer. Measurement of body weight was performed within 0.5 kg using a standard scale placed on a hard flat surface with light clothing and without shoes. Body mass index (BMI) was calculated by dividing weight in kg by the square of height in metres. We used BMI categories applicable to Asian Indians to determine the obesity status (underweight if BMI < 18.5, normal weight if BMI 18.5-22.9, overweight if BMI 23.0-26.9 and obese if BMI ≥ 27.0) (20). Blood pressure was measured following the standard protocol of the Eighth Joint National Committee (JNC-8) guidelines, and hypertension was defined accordingly (21).

Diabetes-related information, such as the duration of DM, the type of diabetes medications, and the presence of diabetes complications, was documented by interviewing and examining the patients. A recent (within the preceding month) HbA1c report was used to determine the glycaemic control of the patients. An HbA1c < 7% was defined as controlled DM, and HbA1c ≥ 7% was defined as uncontrolled DM. This cut-off value is used widely among diabetes patients in Bangladesh (22-24). Reports of the most recent fasting lipid profile were retrieved from participants’ medical records, and dyslipidaemia was defined according to cut-offs described in the Adult Treatment Panel (ATP) III guidelines (25).

#### Assessment of female sexual dysfunction

Female sexual function was assessed with a detailed 19-item questionnaire, the Female Sexual Function Index (FSFI), which was developed by Rosen and cols. (26). This self-reported scale was used to evaluate the six domains of sexual activity, including desire (2 items), arousal (4 items), lubrication (4 items), orgasm (3 items), pain (3 items), and sexual satisfaction (3 items) (26,27). A five-point Likert scale was used to score all the domains. The total score of each domain is multiplied by a certain factor. The factor for desire is 0.6, while it is 0.3 for arousal and lubrication and 0.4 for other domains. In general, each domain has a minimum score of 0-1.2/1.8 and a maximum score of 6. The total score is obtained from the sum of the scores of all the domains and ranges from 2 to 36. Sexual dysfunction was defined as a total FSFI score < 26.55. This is the optimal cut-off score to clinically detect female dysfunction with a sensitivity and specificity of 71% and 88%, respectively (27). The cut-off scores to determine the presence of difficulties in particular domains of the FSFI are as follows: less than 4.28 in the desire domain, less than 5.08 in the arousal domain, less than 5.45 in the lubrication domain, less than 5.05 in the orgasm domain, less than 5.04 in the satisfaction domain, and less than 5.51 in the pain domain (27). In the present study, the Bangla version of the FSFI was used, which was not formally validated for the present study but was previously used among the female population of Bangladesh (28,29). In the present study, the scale showed good internal consistency (Cronbach's alpha 0.78).

### Statistical analysis

All statistical analyses were carried out using SPSS version 26.0. Categorical variables were represented as frequency distributions with percentages, and continuous variables were represented as the means with standard deviations (SDs). An independent t test was used to determine the differences in FSFI domain scores between patients with diabetes and control subjects without diabetes, while the chi-square test was used to determine the difference in the prevalence of sexual dysfunction between these two groups. A binary logistic regression model was used to determine the factors associated with sexual dysfunction among diabetes patients. The results were interpreted with a 95% confidence interval (CI), and a result for which p < 0.05 was considered statistically significant.

## RESULTS

### Characteristics of the participants

A total of 150 T2DM patients and 100 healthy women without diabetes were included in the study. The average age of the patients with diabetes was 35 years (SD 6 years), and they were married for 16.3 years on average, while the age of their counterparts was 30 years (SD 7 years), and they were married for 7.8 years on average. Obesity and comorbid hypertension were more prevalent among the patients with diabetes than among the healthy women without diabetes. Detailed sociodemographic characteristics of the patients and comparison group are described in Table 1.

**Table 1:** Sociodemographic characteristics of T2DM patients and women without diabetes

Variable		Women with T2DM n (%)	Women without diabetes n (%)	p value
Age of the participant		34.8 (5.9)	29.9 (6.9)	<0.001
Age of the participant's husband		42.8 (7.7)	35.5 (9.0)	<0.001
Duration of marriage		16.2 (7.4)	7.7 (6.8)	<0.001
Residence				
	Urban	88 (58.6)	37 (37.0)	0.003
	Suburban	37 (24.6)	36 (36.0)	
	Rural	25 (16.6)	27 (27.0)	
Number of children				
	No child	10 (6.6)	35 (35.0)	<0.001
	1-2	76 (50.6)	53 (53.0)	
	>2	64 (42.6)	12 (12.0)	
Education				
	Self-taught	9 (6.0)	29 (29.0)	<0.001
	Primary	22 (14.6)	16 (16.0)	
	Secondary	65 (43.3)	14 (14.0)	
	Higher secondary	36 (24.0)	8 (8.0)	
	Graduate & above	18 (12.0)	33 (33.0)	
Family type				
	Nuclear	125 (83.3)	34 (34.0)	<0.001
	Joint	25 (16.6)	66 (66.0)	
Employment				
	Homemaker	134 (89.3)	64 (64.0)	<0.001
	Office job	16 (10.6)	36 (36.0)	
Menstrual cycle				
	Regular	48 (32.0)	75 (75.0)	0.233
	Irregular	102 (68.0)	25 (25.0)	
Body mass index (BMI)				
	Normal	23 (15.3)	30 (30.0)	<0.001
	Underweight	4 (2.6)	17 (17.0)	
	Overweight	72 (48.0)	46 (46.0)	
	Obese	51 (34.0)	7 (7.0)	
Comorbid hypertension (HTN)				
	No	67 (44.6)	71 (71.0)	0.013
	Yes	83 (55.3)	29 (29.0)	

T2DM: type 2 diabetes mellitus.

### Prevalence of sexual dysfunction

The overall prevalence of sexual dysfunction was higher in women with diabetes than in women without diabetes (79% *vs.* 72%). The mean FSFI score was significantly lower among the diabetes patients (mean 20.86, SD 7.26) than among the women without diabetes (mean 23.77, SD 4.80). Scores of individual domains, except arousal, were also significantly lower among the diabetes patients (Table 2). Domain-wise dysfunctions were also similar between the two groups after adjustment for age (Table 3).

**Table 2:** FSFI scores in the participants with and without diabetes

FSFI domains	Women with T2DM Mean (SD)	Women without diabetes Mean (SD)	p value
Desire Score	2.9 (1.0)	3.2 (1.1)	0.046
Arousal Score	3.1 (1.3)	3.3 (0.9)	0.092
Lubrication Score	3.6 (1.3)	3.9 (0.8)	0.015
Orgasm Score	3.5 (1.4)	3.9 (1.0)	0.010
Satisfaction Score	3.6 (1.4)	4.3 (1.1)	<0.001
Pain Score	3.9 (1.6)	4.8 (1.0)	<0.001
Total FSFI Score	20.8 (7.2)	23.7 (4.8)	<0.001

FSFI: Female Sexual Function Index; T2DM: type 2 diabetes mellitus; SD: standard deviation.

**Table 3:** Prevalence of sexual dysfunction among T2DM patients and controls

FSFI domains		Women with T2DM, n (%)	Women without diabetes, n (%)	aOR (95% CI)	p value
Overall Sexual Dysfunction		119 (79.3)	72 (72.0)	1.05 (0.55-2.02)	0.864
Domain wise dysfunction					
	Desire Problem	140 (93.3)	86 (86.0)	1.33 (0.52-3.43)	0.548
	Arousal Problem	141 (94.0)	92 (92.0)	0.89 (0.31-2.64)	0.835
	Lubrication Problem	146 (97.3)	97 (97.0)	1.01 (0.19-5.15)	0.997
	Orgasm Problem	133 (88.7)	83 (83.0)	1.27 (0.58-2.79)	0.547
	Satisfaction Problem	128 (85.3)	75 (75.0)	1.38 (0.69-2.78)	0.359
	Pain Problem	123 (82.0)	67 (67.0)	2.85 (1.49-5.42)	0.007

aOR: odds ratio for T2DM patients adjusted for age; FSFI: Female Sexual Function Index; T2DM: type 2 diabetes mellitus; CI: confidence interval.

### Predictors of sexual dysfunction among T2DM patients

In a bivariate analysis, it was found that the duration of diabetes and the level of HbA1c were associated with sexual dysfunction among female patients with diabetes. Moreover, sexual dysfunction was more prevalent among patients with comorbid hypertension and diabetes complications (Table 4).

**Table 4:** Characteristics of T2DM patients according to sexual dysfunction

Characteristics			Total	Sexual dysfunction		
				Present	Absent	p value
Age			33.4 (5.8)	35.2 (5.9)	33.4 (5.9)	0.073
Husbands’ age			40.6 (7.0)	43.4 (7.8)	40.6 (7.2)	0.069
Marriage duration			14.4 (7.4)	16.7 (7.4)	14.4 (7.4)	0.060
Diabetes duration			3.5 (2.8)	5.8 (4.5)	3.5 (2.8)	0.004
HbA1c			7.5 (1.2)	8.3 (1.9)	7.5 (1.2)	0.013
	Residence					
		Urban	88 (58.6)	69 (78.4)	19 (21.5)	0.718
		Suburban	37 (24.6)	31 (83.7)	6 (16.2)	
		Rural	25 (16.6)	19 (76.0)	6 (24.0)	
Number of children						
	0		10 (6.6)	8 (80.0)	2 (20.0)	0.618
	1-2		76 (50.6)	58 (76.3)	18 (23.6)	
	>2		64 (42.6)	53 (82.8)	11 (17.2)	
Education						
	Self-learned		9 (6.0)	7 (77.7)	2 (22.2)	
	Primary		22 (14.6)	20 (90.9)	2 (9.1)	0.205
	Secondary		65 (43.3)	54 (83.0)	11 (16.9)	
	Higher Secondary		36 (24.0)	24 (66.6)	12 (33.3)	
	Graduate & above		18 (12.0)	14 (77.7)	4 (22.2)	
Family type						
	Nuclear		125 (83.3)	98 (78.4)	27 (21.6)	0.528
	Joint		25 (16.6)	21 (84.0)	4 (16.0)	
Employment						
	Home maker		134 (89.3)	108 (80.6)	26 (19.4)	0.269
	Office job		16 (10.6)	11 (68.7)	5 (31.2)	
Menstruation						
	Irregular		48 (32.0)	39 (81.2)	9 (18.7)	0.691
	Regular		102 (68.0)	80 (78.4)	22 (21.5)	
HTN						
	Yes		67 (44.6)	58 (86.5)	9 (13.4)	0.049
	No		83 (55.3)	61 (73.4)	22 (26.5)	
BMI						
	Normal		23 (15.3)	15 (65.2)	8 (34.7)	0.083
	Underweight		4 (2.6)	4 (100.0)	0 (0.0)	
	Overweight		72 (48.0)	55 (76.3)	17 (23.6)	
	Obese		51 (34.0)	45 (88.2)	6 (11.7)	
Diabetes complications						
	Yes		54 (36.0)	49 (90.7)	5 (9.2)	0.010
	No		96 (64.0)	70 (72.9)	26 (27.1)	

FSFI: Female Sexual Function Index; T2DM: type 2 diabetes mellitus; HTN: hypertension; BMI: body mass index.

Multiple logistic regression models, which were performed to identify the predictors of the different domains of sexual dysfunction, revealed that the duration of diabetes was only associated with orgasm problems (aOR 1.33, 95% CI 1.01-1.76) and pain during intercourse (aOR 1.26, 95% CI 1.01-1.56). In addition, patients with obesity showed a greater risk of having painful coitus (aOR 9.53, 95% CI 1.77-51.33) (Table 5).

**Table 5:** Predictors of sexual dysfunction and its domains (logistic regression model)

Variables	FSD	Desire	Arousal	Lubrication	Orgasm	Satisfaction	Pain
Patient's age	0.91	1.26	0.95	0.64	0.85	0.97	0.91
	(0.77-1.07)	(0.95-1.66)	(0.69-1.31)	(0.34-1.23)	(0.68-1.06)	(0.81-1.16)	(0.77-1.09)
Husband's age	1.05	0.92	0.97	1.16	1.12	1.01	1.12
	(0.92-1.19)	(0.75-1.12)	(0.75-1.26)	(0.75-1.79)	(0.92-1.35)	(0.87-1.16)	(0.98-1.29)
Duration of marriage	0.98	0.91	0.98	1.23	0.96	1.03	0.89
	(0.88-1.10)	(0.74-1.12)	(0.80-1.28)	(0.75-2.01)	(0.83-1.10)	(0.91-1.16)	(0.79-1.01)
Menstruation	1.21	1.56	0.30	0.04	1.01	1.21	0.48
	(0.47-3.13)	(0.29-8.38)	(0.06-1.47)	(0.01-1.45)	(0.28-3.55)	(0.42-3.53)	(0.18-1.29)
Duration of DM	1.19	0.91	1.33	1.39	1.33	1.11	1.26
	(0.99-1.44)	(0.67-1.22)	(0.81-2.18)	(0.68-2.85)	(1.01-1.76)*	(0.91-1.36)	(1.01-1.56)*
HTN (Yes)	1.10	2.01	1.94	2.77	3.63	0.91	0.99
	(0.37-3.24)	(0.27-14.48)	(0.18-20.58)	(0.82-4.07)	(0.67-9.59)	(0.27-3.08)	(0.31-3.20)
BMI							
Normal	Ref.	Ref.	Ref.	Ref.	Ref.	Ref.	Ref.
Overweight	1.52	1.71	2.14	0^a^	0.49	0.54	2.91
	(0.51-4.46)	(0.36-7.99)	(0.41-11.12)		(0.10-2.62)	(0.13-2.18)	(0.94-8.95)
Obese	3.02	4.30	0^b^	0^a^	0.96	0.84	9.53
	(0.72-12.63)	(0.38-47.93)			(0.11-8.39)	(0.15-4.58)	(1.77-51.33)*
HbA1c	1.15	1.34	1.18	1.14	0.80	1.09	1.01
	(0.83-1.60)	(0.65-2.77)	(0.57-2.43)	(0.47-2.76)	(0.55-1.15)	(0.77-1.54)	(0.73-1.39)
Diabetes complication	1.81	2.95	0^b^	0.15	2.17	1.75	0.88
	(0.54-6.01)	(0.23-36.99)		(0.03-6.77)	(0.37-12.73)	(0.46-6.66)	(0.25-3.08)

a Omitted due to collinearity problem.

b Empty cell.

* p value < 0.05

FSD: Female sexual dysfunction; DM: diabetes mellitus; HTN: hypertension; BMI: body mass index.

## DISCUSSION

The present study provides baseline evidence on the prevalence of sexual dysfunction among women with diabetes in Bangladesh. Our results demonstrated that patients with diabetes mellitus scored significantly lower on the indices of the FSFI, except for the arousal index, compared to patients without diabetes. A similar phenomenon was also observed in different studies that reported that the mean FSFI indices and the global scores were lower in the group of individuals with diabetes than in the group of individuals without diabetes (6,30-32).

According to our findings, a total of 79% of female patients with diabetes and 72% of women without diabetes suffered from sexual dysfunction. Among the domains of sexual dysfunction, problems related to lubrication (97%), arousal (94%), and decreased desire (93%) were the most prevalent in women with diabetes, followed by problems related to orgasm (89%), satisfaction (85%), and pain during intercourse (82%). The prevalence was mostly similar in the women with and without diabetes, except for pain during intercourse. However, these findings should be interpreted with caution, as the patients with T2DM and the healthy control group were not matched for the baseline characteristics, and hence, there were other differences between them. Females in the T2DM group were older than those in the control group, and their duration of conjugal life was also longer, which might impair their sexual relations.

A very large discrepancy in the prevalence of sexual dysfunction among female patients with diabetes has been reported in the literature. However, there is little evidence on this issue among women with diabetes in Bangladesh to compare our findings. Few studies conducted among female patients attending psychiatric or gynaecological outpatient departments reported that approximately 54% of them suffered from sexual dysfunction, which is comparatively lower than our findings, even from women without diabetes (5,28). The findings of our study corroborate a study from Iran, China and Nigeria, where the prevalence was 78%, 79% and 71%, respectively (30,33,34). A study from neighbouring India reported that the prevalence of sexual dysfunction was 32% among women with diabetes, which is also much lower than our finding (35).

Our study revealed little difference in the prevalence of sexual dysfunction between participants with and without diabetes. Some studies, such as Ammar and cols. (17) and Shi and cols. (30) reported that the prevalence was significantly higher among women with diabetes than among women without diabetes. On the other hand, some studies, such as Ezeani and cols., conducted among Nigerian women with diabetes reported no difference in the prevalence between participants with and without diabetes (34). However, a recent study conducted among Egyptian women reported that women with T2DM had a higher prevalence of sexual dysfunction than healthy controls (36). A high prevalence of different domains of sexual dysfunction, including decreased sexual desire, problems in arousal and dysfunction and pain during coitus, was also reported by some studies (34,37,38), reflecting our findings.

Neurovascular damage due to diabetes mellitus impairs the nervous response to erotic stimuli, resulting in decreased sexual desire, arousal, and satisfaction. Moreover, decreased genital blood flow, endothelial damage, and persistent hyperglycaemia may impair vaginal lubrication and increase the chance of infection, resulting in pain during intercourse (12,13). However, studies in different countries have shown a large disparity in the prevalence of sexual disorders (6). As a subjective measurement, domains of sexual dysfunction may be perceived differently by individual patients. Moreover, the prevalence may be different according to the characteristics of the measuring tools. In addition, patients’ personal beliefs, perceptions and sociocultural structure may influence the prevalence. A decreasing trend in the prevalence of sexual dysfunction was also observed with the increasing sample size included in the study (6).

In our study, no diabetes-related factors were found to be associated with sexual dysfunction among female patients. However, diabetes duration was associated with painful coitus and problematic orgasm. Sexual pain was also found to be a component of impaired sexual satisfaction among female patients with diabetes in a previous study (38). Attempts to identify the risk factors for sexual dysfunction among women with diabetes have shown ambiguous and inconclusive findings. Few studies have reported age, obesity, glycaemic control, diabetes complications, and comorbid hypertension as predictors of female sexual dysfunction (34,35,37-40). In contrast, most of the studies indicated poor or no association between sexual dysfunction and diabetes-related factors, such as glycaemic control or duration of diabetes, as well as other parameters, such as age, obesity, menstrual characteristics, or use of hormonal contraceptives or replacement therapy, which corroborates our findings (15,33,35,41). Moreover, it was reported that sexual function decreases during the luteal phase in comparison with the follicular phase in women, especially those with T1DM, although sexual function remains similar during both phases in women with T2DM (42). However, female sexual function in diabetes is influenced significantly by psychological distress, such as depression and diabetes stress (12,41,43).

### Limitations

The present study is one of the first investigations to identify the prevalence and associated factors of sexual dysfunction among Bangladeshi women with T2DM. Despite this fact, it has several limitations. First, as a facility-based study, only patients with diabetes who visited the selected hospital were included. Therefore, the findings cannot be inferential for the overall patient population of the country. The temporal relationship between different factors and sexual dysfunction could not be established in this study design. In addition, we did not include potential psychological risk factors for sexual dysfunction in females, such as depression and stress, which could make our findings inconclusive. Finally, as the topic of the study was a culturally sensitive and embarrassing issue for the comparatively conservative society of Bangladesh, social desirability bias could not be avoided.

In conclusion, sexual dysfunction among women with diabetes often remains a neglected issue in diabetes management. Our study found that its prevalence was quite high among the study population. Obesity, longstanding diabetes, and high HbA1c levels were associated with sexual dysfunction. The issue of sexual health should be included in the diabetes management plan, and health care providers should address the issue in their routine discussions with diabetes patients.
